# Reply to Jalink, M.B. Comment on “Senthilkumaran et al. Bilateral Simultaneous Optic Neuritis Following Envenomations by Indian Cobra and Common Krait. *Toxins* 2022, *14*, 805”

**DOI:** 10.3390/toxins16030115

**Published:** 2024-02-27

**Authors:** Subramanian Senthilkumaran, Stephen W. Miller, Harry F. Williams, Ponniah Thirumalaikolundusubramanian, Ketan Patel, Sakthivel Vaiyapuri

**Affiliations:** 1 Manian Medical Centre, Erode 638001, Tamil Nadu, India; maniansenthil76@gmail.com; 2 The Poison Control Center, Children’s Hospital of Philadelphia, Philadelphia, PA 19104, USA; stephenmiller@isu.edu; 3 Toxiven Biotech Private Limited, Coimbatore 641042, Tamil Nadu, India; harry@toxiven.com; 4 Department of General Medicine, The Tamil Nadu Dr. M.G.R Medical University, Chennai 600032, Tamil Nadu, India; ponniah.tks@gmail.com; 5 School of Biological Sciences, University of Reading, Reading RG6 6UB, UK; ketan.patel@reading.ac.uk; 6 School of Pharmacy, University of Reading, Reading RG6 6UB, UK

We thank the author for showing interest in our article [1] and proposing antivenom as a potential cause for the snakebite-induced bilateral optic neuritis that we reported in two snakebite victims [2]. Our primary aim of this article was to report the “polo mint” appearance that we observed using MRI in those patients and to emphasise the necessity for early diagnosis using sophisticated techniques such as MRI and treatment with corticosteroids to promptly tackle this condition. Moreover, to the best of our knowledge, krait bite-induced optic neuritis has not been reported previously, and thus, we reported the first case of krait bite developing this condition. Through this article, we also wanted to caution clinicians that they should continue to monitor patients for any delayed complications such as optic neuritis including in krait envenomation. They should consider using techniques such as MRI to identify the precise damage and use corticosteroids promptly to manage this condition. This condition can be caused by various issues including bacterial and viral infections [3,4]. Optic neuritis and similar manifestations have been previously reported in some viper [5,6,7] and elapid [8] snakebite victims. However, the underlying molecular mechanisms that induce optic neuritis in snakebite victims were not completely understood. We highlighted a list of potential causes for snakebite-induced optic neuritis in our article including snakebite-induced haemorrhage and inflammation, venom, and antivenom. As the precise mechanisms for optic neuritis following snakebite envenomation are largely unknown, further research is essential to establish the molecular relationships between snakebites/venoms and optic neuritis. Hence, we agree with the author that antivenom could be a potential cause along with several other factors for optic neuritis following envenoming. Further scientific research could establish the causes and underlying mechanisms for developing optic neuritis in snakebite victims.
